# Supplementary material for: Non-linear age dynamics of malaria infection and fine-scale environmental exposure in rural Uganda
Source: BMC Med. 2026 Jun 19;24:523. doi: 10.1186/s12916-026-04928-3 (PMC13617724; doi:10.1186/s12916-026-04928-3)
Supplement: Supplementary file 1 [file 12916_2026_4928_MOESM5_ESM.pdf]

STROBE Statement—checklist of items that should be included in reports of observational studies

	Item No.	Recommendation	Page No.	Relevant text from manuscript
**Title and abstract**	1	(*a*) Indicate the study's design with a commonly used term in the title or the abstract	1-2	See title
		(*b*) Provide in the abstract an informative and balanced summary of what was done and what was found	1-2	See abstract
		**Introduction**		
Background/rationale	2	Explain the scientific background and rationale for the investigation being reported	2-3	Paragraph 1-3 provide background and paragraph 4 explains the rationale
Objectives	3	State specific objectives, including any prespecified hypotheses	3	Paragraph 4
		**Methods**		
Study design	4	Present key elements of study design early in the paper	3-4	See Study design and participants
Setting	5	Describe the setting, locations, and relevant dates, including periods of recruitment, exposure, follow-up, and data collection	3-4	See Study design and participants
Participants	6	(*a*) *Cohort study*—Give the eligibility criteria, and the sources and methods of selection of participants. Describe methods of follow-up<br>*Case-control study*—Give the eligibility criteria, and the sources and methods of case ascertainment and control selection. Give the rationale for the choice of cases and controls<br>*Cross-sectional study*—Give the eligibility criteria, and the sources and methods of selection of participants	3-4	See Study design and participants
		(*b*) *Cohort study*—For matched studies, give matching criteria and number of exposed and unexposed<br>*Case-control study*—For matched studies, give matching criteria and the number of controls per case	---	---
Variables	7	Clearly define all outcomes, exposures, predictors, potential confounders, and effect modifiers. Give diagnostic criteria, if applicable	4-5	See Malaria outcomes, Environmental exposure, Sociodemographic and health access covariates, as well as

				supplementary S2 Text: Covariate Definitions and Categorizations
Data sources/ measurement	8*	For each variable of interest, give sources of data and details of methods of assessment (measurement). Describe comparability of assessment methods if there is more than one group	*4-5*	See Study design and participants
Bias	9	Describe any efforts to address potential sources of bias	5-6	See Statistical Analysis
Study size	10	Explain how the study size was arrived at	3-4	See Study design and participants

Continued on next page

Quantitative variables	11	Explain how quantitative variables were handled in the analyses. If applicable, describe which groupings were chosen and why	5-6	See Malaria Outcomes, Environmental Exposure, Sociodemographic and health access covariates
Statistical methods	12	(*a*) Describe all statistical methods, including those used to control for confounding	6-7	See Section Statistical Analysis and Supplement and S4 Text
		(*b*) Describe any methods used to examine subgroups and interactions	6-7	See Section Statistical Analysis and Supplement and S4 Text
		© Explain how missing data were addressed	4-7	See Section Statistical Analysis and Supplement and S4 Text
		(*d*) *Cohort study*—If applicable, explain how loss to follow-up was addressed<br>*Case-control study*—If applicable, explain how matching of cases and controls was addressed<br>*Cross-sectional study*—If applicable, describe analytical methods taking account of sampling strategy	6-7	See Section Statistical Analysis and Supplement and S4 Text
		(*e*) Describe any sensitivity analyses	6-7	See Section Statistical Analysis and Supplement and S4 Text
**Results**				
Participants	13*	(a) Report numbers of individuals at each stage of study—eg numbers potentially eligible, examined for eligibility, confirmed eligible, included in the study, completing follow-up, and analysed	7-8	See Malaria Status and parasitaemia, Table 2 and 3
		(b) Give reasons for non-participation at each stage	7	See Malaria Status and parasitaemia
		(c) Consider use of a flow diagram	-	-
Descriptive data	14*	(a) Give characteristics of study participants (eg demographic, clinical, social) and information on exposures and potential confounders	8-10	See Table 3
		(b) Indicate number of participants with missing data for each variable of interest	7-10	See Table 3
		© *Cohort study*—Summarise follow-up time (eg, average and total amount)	-	
Outcome data	15*	*Cohort study*—Report numbers of outcome events or summary measures over time	-	
		Case-control study—Report numbers in each exposure category, or summary measures of exposure	-	
		Cross-sectional study—Report numbers of outcome events or summary measures	*7-10*	See Table and Malaria Status
Main results	16	(*a*) Give unadjusted estimates and, if applicable, confounder-adjusted estimates and their precision (eg, 95% confidence interval). Make clear which confounders were adjusted for and why they were included	15-17	See *Model performance and sensitivity analyses*, Figure 4, 5, and

			6, Supplement Figures S2-S4, S7-S13
	(*b*) Report category boundaries when continuous variables were categorized	15-17	See *Non-linear age dynamics of malaria infection* and section *Sociodemographic, environmental, and health access determinants of malaria*
	© If relevant, consider translating estimates of relative risk into absolute risk for a meaningful time period	-	-

Continued on next page

Other analyses	17	Report other analyses done—eg analyses of subgroups and interactions, and sensitivity analyses	6-21	See *Model performance and sensitivity analyses* and Supplementary Material
**Discussion**				
Key results	18	Summarise key results with reference to study objectives	18	Discussion, first paragraph
Limitations	19	Discuss limitations of the study, taking into account sources of potential bias or imprecision. Discuss both direction and magnitude of any potential bias	23	Discussion, last paragraph paragraph
Interpretation	20	Give a cautious overall interpretation of results considering objectives, limitations, multiplicity of analyses, results from similar studies, and other relevant evidence	18-23	See discussion paragraphs 2-8
Generalisability	21	Discuss the generalisability (external validity) of the study results	18-23	Discussion, last paragraph paragraph
**Other information**				
Funding	22	Give the source of funding and the role of the funders for the present study and, if applicable, for the original study on which the present article is based	25	See Funding Declaration

*Give information separately for cases and controls in case-control studies and, if applicable, for exposed and unexposed groups in cohort and cross-sectional studies.

Note: An Explanation and Elaboration article discusses each checklist item and gives methodological background and published examples of transparent reporting. The STROBE checklist is best used in conjunction with this article (freely available on the Web sites of PLoS Medicine at http://www.plosmedicine.org/, Annals of Internal Medicine at http://www.annals.org/, and Epidemiology at http://www.epidem.com/). Information on the STROBE Initiative is available at www.strobe-statement.org.
