# Supplementary material for: Non-linear age dynamics of malaria infection and fine-scale environmental exposure in rural Uganda
Source: BMC Med. 2026 Jun 19;24:523. doi: 10.1186/s12916-026-04928-3 (PMC13617724; doi:10.1186/s12916-026-04928-3)
Supplement: Supplementary file 3 [file 12916_2026_4928_MOESM2_ESM.docx]

# Additional File 2: Supplementary Tables S1 – S7

| Table S1: | Comprehensive summary of Sentinel-2 data availability and quality. |
| --- | --- |
| Table S2: | Environmental indices by water body type at malacological survey sites. |
| Table S3: | Household occupancy by number of rooms. |
| Table S4: | Proportion of RDT-positive participants reporting fever within the past month by antimalarial treatment status. |
| Table S5: | Effect of occupation on malaria infection with and without adjustment for age and gender. |
| Table S6: | Cross-validated confusion matrix and performance metrics for the final malaria prediction model. |
| Table S7: | Comparison of Intra-class Correlation Coefficient (ICC) Across Nested Models. |

**Table S1**: Comprehensive summary of Sentinel-2 data availability and quality. Each analysis period spans from December 1 to January 31. Despite high average cloud cover in the source imagery, the median composite method produced a cloud-free mosaic with no excluded pixels.

| District | Analysis Period | Available Scenes | Cloud Cover (%) | Total Pixels | Pixels Used | Excl. Pixels |
| --- | --- | --- | --- | --- | --- | --- |
| Pakwach | 2021–2022 | 23 | 43.10 | 2915941 | 2915941 | 0 |
| Buliisa | 2021–2022 | 48 | 43.45 | 3099069 | 3099069 | 0 |
| Mayuge | 2021–2022 | 9 | 58.19 | 5151172 | 5151172 | 0 |
| Pakwach | 2022–2023 | 22 | 30.88 | 2915941 | 2915941 | 0 |
| Buliisa | 2022–2023 | 44 | 36.26 | 3099069 | 3099069 | 0 |
| Mayuge | 2022–2023 | 12 | 55.74 | 5151172 | 5151172 | 0 |
| Pakwach | 2023–2024 | 24 | 43.76 | 2915941 | 2915941 | 0 |
| Buliisa | 2023–2024 | 48 | 48.62 | 3099069 | 3099069 | 0 |
| Mayuge | 2023–2024 | 11 | 58.77 | 5151172 | 5151172 | 0 |

**Table S2**: Environmental indices by water body type at malacological survey sites.

Index	Overall	Lake marsh	Lake beach	Pond/swamp	Rice paddy	River/marsh (*n* = 437)	(*n* = 162)	(*n* = 151)	(*n* = 60)	(*n* = 9)	(*n* = 55)

**NDVI**

**EVI**

**NDWI**

**MNDWI**

Mean	0*.*41	0*.*48	0*.*20	0*.*57	0*.*63	0*.*57

SD	0*.*22	0*.*15	0*.*14	0*.*18	0*.*05	0*.*14

Median	0*.*44	0*.*51	0*.*17	0*.*64	0*.*63	0*.*61

IQR	[0.22, 0.61]	[0.37, 0.61]	[0.09, 0.28]	[0.52, 0.69]	[0.60, 0.66]	[0.51, 0.68]

Mean	0*.*26	0*.*30	0*.*13	0*.*37	0*.*39	0*.*36

SD	0*.*14	0*.*10	0*.*08	0*.*12	0*.*04	0*.*10

Median	0*.*27	0*.*32	0*.*12	0*.*41	0*.*39	0*.*39

IQR	[0.14, 0.38]	[0.23, 0.38]	[0.07, 0.19]	[0.33, 0.45]	[0.36, 0.41]	[0.28, 0.44]

Mean	*−*0*.*36	*−*0*.*42	*−*0*.*17	*−*0*.*52	*−*0*.*57	*−*0*.*49

SD	0*.*20	0*.*14	0*.*13	0*.*15	0*.*04	0*.*13

Median	*−*0*.*39	*−*0*.*46	*−*0*.*16	*−*0*.*58	*−*0*.*58	*−*0*.*54

IQR	[*−*0.55, *−*0.19]	[*−*0.54, *−*0.32]	[*−*0.24, *−*0.08]	[*−*0.60, *−*0.49]	[*−*0.61, *−*0.57]	[*−*0.58, *−*0.44]

Mean	*−*0*.*25	*−*0*.*30	*−*0*.*12	*−*0*.*38	*−*0*.*42	*−*0*.*33

SD	0*.*16	0*.*13	0*.*13	0*.*11	0*.*06	0*.*10

Median	*−*0*.*28	*−*0*.*32	*−*0*.*13	*−*0*.*41	*−*0*.*44	*−*0*.*36

IQR	[*−*0.39, *−*0.14]	[*−*0.40, *−*0.23]	[*−*0.20, *−*0.06]	[*−*0.46, *−*0.37]	[*−*0.47, *−*0.38]	[*−*0.40, *−*0.29]

31

**Table S3**: Household occupancy by number of rooms.

Number of	Number of People	Sample

| Rooms | Mean (SD) | Median | IQR | Size (n) |
| --- | --- | --- | --- | --- |
| 1 | 3.2 (1.1) | 3.0 | [3, 4] | 1,286 |
| 2 | 3.7 (1.4) | 3.0 | [3, 5] | 1,878 |
| 3 | 4.0 (1.7) | 4.0 | [3, 5] | 703 |
| 4 | 4.4 (1.7) | 4.0 | [3, 5] | 269 |
| 5 | 4.1 (1.6) | 4.0 | [3, 5] | 82 |
| 6+ | 4.1 (1.7) | 4.0 | [3, 5] | 90 |
| Total |  |  |  | 4,308 |

**Table S4**: Proportion of RDT-positive participants reporting fever within the past month by antimalarial treatment status among 1431 individuals from 2023-2024, stratified by age group.

| Age Group |  | Antimalarial Status | |  |
| --- | --- | --- | --- | --- |
|  |  | Treated | Not Treated | p-value |
| 5 to *<*11 years | Fever | 57 (34.1%) | 24 (25.0%) | 0.160 |
|  | No Fever | 110 (65.9%) | 72 (75.0%) |  |
| 11 to *<*20 years | Fever | 25 (25.5%) | 17 (18.1%) | 0.285 |
|  | No Fever | 73 (74.5%) | 77 (81.9%) |  |
| *≥* 20 years | Fever | 4 (5.6%) | 16 (14.8%) | 0.096 |
|  | No Fever | 67 (94.4%) | 92 (85.2%) |  |

**Table S5**: Effect of occupation on malaria infection with and without adjust-ment for age and gender.

**Model	Variable	Estimate	SE	z value	p-value**

*Model 1: Occupation only (Deviance explained = 11.3%)*

Intercept	*−*0*.*062	0*.*100	*−*0*.*619	0.536

Farmer	*−*1*.*458	0*.*106	*−*13*.*704	*<* 0*.*001

Fisherman	*−*1*.*032	0*.*141	*−*7*.*315	*<* 0*.*001

Fishmonger	*−*1*.*270	0*.*231	*−*5*.*495	*<* 0*.*001

*Model 2: Occupation + Age (Deviance explained = 19.8%)*

Intercept	*−*0*.*435	0*.*115	*−*3*.*785	< 0.001

Farmer	*−*0*.*205	0*.*128	*−*1*.*609	0.108

Fisherman	0*.*248	0*.*160	1*.*550	0.121

Fishmonger	0*.*038	0*.*243	0*.*155	0.877

Age (edf = 6.3)	–	–	–	< 0.001

*Model 3: Occupation + Gender (Deviance explained = 12.3%)*

Intercept	0*.*230	0*.*106	2*.*164	0.031

Female	*−*0*.*547	0*.*071	*−*7*.*704	*<* 0*.*001

Farmer	*−*1*.*360	0*.*108	*−*12*.*635	*<* 0*.*001

Fisherman	*−*1*.*316	0*.*146	*−*9*.*014	*<* 0*.*001

Fishmonger	*−*1*.*091	0*.*232	*−*4*.*697	*<* 0*.*001

*Model 4: Occupation + Age + Gender (Deviance explained = 20.0%)*

Intercept	*−*0*.*276	0*.*122	−2.262	0.024

Female	*−*0*.*279	0*.*077	−3.652	< 0.001

Farmer	*−*0*.*189	0*.*128	*−*1*.*477	0.140

Fisherman	0*.*064	0*.*167	0*.*386	0.700

Fishmonger	0*.*092	0*.*244	0*.*379	0.705

Age (edf = 6.1)	–	–	–	< 0.001

Note: All models include village random effects. Reference category for occupation is none/other.

**Table S6**: Cross-validated confusion matrix and per-formance metrics for the final malaria prediction model.

Reference

| Predicted | Negative (0) | Positive (1) | Total |
| --- | --- | --- | --- |
| Negative (0) | 1807 | 481 | 2288 |
| Positive (1) | 725 | 1295 | 2020 |
| Total | 2,532 | 1,776 | 4,308 |

| Performance Metric | Value (95% CI) |  |
| --- | --- | --- |
| Accuracy | 0.7201 |  |
| Sensitivity | 0.7292 |  |
| Specificity | 0.7137 |  |
| Positive Predictive Value | 0.6411 |  |
| Negative Predictive Value | 0.7898 |  |
| Balanced Accuracy | 0.7214 |  |
| Cohen’s Kappa | 0.4339 |  |

**Table S7**: Comparison of Intra-class Correlation Coefficient (ICC) Across Nested Models.

| **Model** | *NObs* | *NVillages* | **Village Var. (***σ*^2^**)** | **ICC** |
| --- | --- | --- | --- | --- |
| Intercept + Village Random Effect | 4,308 | 52 | 0.3087 | 0.0858 |
| Base Model + Village Random Effect | 4,308 | 52 | 0.2543 | 0.0717 |
| NDVI Only + Village Random Effect | 4,308 | 52 | 0.2420 | 0.0685 |
| MNDWI Only + Village Random Effect | 4,308 | 52 | 0.1008 | 0.0297 |
| Base + NDVI + Village Random Effect | 4,308 | 52 | 0.2081 | 0.0595 |
| Base + MNDWI + Village Random Effect | 4,308 | 52 | 0.1664 | 0.0482 |
| Base + NDVI + MNDWI + Village Random Effect | 4,308 | 52 | 0.1556 | 0.0452 |
| **Main Model (All Predictors)** | **4,308** | **52** | **0.1371** | **0.0400** |
