# Supplementary material for: Non-linear age dynamics of malaria infection and fine-scale environmental exposure in rural Uganda
Source: BMC Med. 2026 Jun 19;24:523. doi: 10.1186/s12916-026-04928-3 (PMC13617724; doi:10.1186/s12916-026-04928-3)
Supplement: Supplementary file 4 [file 12916_2026_4928_MOESM3_ESM.docx]

# Additional File 3: Supplementary Figures S1 to S20

Fig S1: Areal-weighted change-of-support with a pointy-topped hexagon over 10 m pixels.

Fig S2: NDVI time-series for Mayuge district (2021–2025), generated using a 60-day moving window composite.

Fig S3: NDVI time-series for Buliisa district (2021–2025), generated using a 60-day moving window composite.

Fig S4: NDVI time-series for Pakwach district (2021–2025), generated using a 60-day moving window composite.

Fig S5: Community Mapping empty Grid

Fig S6: Community Mapping Symbol Legend

Fig S7: Structure of the adaptive penalty matrices for the age spline across different penalty basis dimensions (*m* = 1*,* 3*,* 5).

Fig S8: Spatial distribution of study sites and NDVI analysis in Mayuge.

Fig S9: Spatial distribution of study sites and NDVI analysis in Pakwach.

Fig S10: Validation of environmental indices against ground truth ecological data for malaco-logical survey sites by Landform),

Fig S11: Validation of environmental indices against ground truth ecological data for malaco-logical survey sites (by Floating Vegetation)

Fig S12: Distribution of Land Surface Temperature (LST) and Normalized Difference Vege-tation Index (NDVI) values across study villages.

Fig S13: Comparison of the within-village coefficient of variation (CV) for Land Surface Tem-perature (LST) and NDVI.

Fig S14: Comparison of model coefficient estimates for models using raw, unsmoothed NDVI (red) versus spatially smoothed NDVI (teal).

Fig S15: Pairwise Spearman’s correlation matrix of all candidate predictor variables.

Fig S16: Receiver operating characteristic (ROC) curve for the final malaria prediction model.

Fig S17: Joint interaction surface of age and environmental exposure.

Fig S18: Modeled joint effect of age and NDVI on malaria infection probability.

Fig S19: Determinants of malaria infection with alternative home quality score.

Fig S20: Determinants of malaria infection of participants from 2023 and 2024 including bed net use.

Square raster (10 m cells)

flat-to-flat *≈ √*3*a ≈* 32*.*2 m

point-to-point *≈* 2*a ≈* 37*.*2 m

Hex cell (*A ≈* 900 m^2^)

10 m

**Fig. S1**: Areal-weighted change-of-support with a pointy-topped hexagon over 10 m pixels. Pixel contributions are weighted by fractional overlap *A_ir_/A_i_*.

**Fig. S2**: NDVI time-series for Mayuge district (2021–2025), generated using a 60-day moving window composite. The highlighted ribbons show the Dec 1 – Jan 31 analytical periods used in the main analysis, corresponding to the seasonal vegetation minimum.

**Fig. S3**: NDVI time-series for Buliisa district (2021–2025), generated using a 60-day moving window composite. The highlighted ribbons show the Dec 1 – Jan 31 analytical periods used in the main analysis, corresponding to the seasonal vegetation minimum.

**Fig. S4**: NDVI time-series for Pakwach district (2021–2025), generated using a 60-day moving window composite. The highlighted ribbons show the Dec 1 – Jan 31 analytical periods used in the main analysis, corresponding to the seasonal vegetation minimum.

**Fig. S5**: Community Mapping empty Grid

**Fig. S6**: Community Mapping Symbol Legend

**Fig. S7**: Structure of the adaptive penalty matrices for the age spline across different penalty basis dimensions (*m* = 1*,* 3*,* 5). Each heatmap visualizes a component penalty matrix (**S***_j_*) scaled by its corresponding estimated smoothing parameter (*λ*ˆ*j* , labeled ’sp’). The final model utilized *m* = 3 (middle row), where the overall penalty is the sum of three such weighted matrices. This allows the wiggliness of the age-response curve to vary flexibly with age, in contrast to a standard smooth with a single penalty (*m* = 1, top row).

**Fig. S8**: Spatial distribution of study sites and NDVI analysis in Mayuge. **(a)** Regional map showing household locations (blue markers). **(b)** Sentinel-2 satellite imagery of the area outlined in (a).

**(c)** Unsmoothed NDVI values. **(d)** Spatially smoothed NDVI used for statistical analysis. The color gradient in (c) and (d) indicates vegetation density, from low (red) to high (green).

**Fig. S9**: Spatial distribution of study sites and NDVI analysis in Pakwach. **(a)** Regional map showing household locations (blue markers). **(b)** Sentinel-2 satellite imagery of the area outlined in (a).

**(c)** Unsmoothed NDVI values. **(d)** Spatially smoothed NDVI used for statistical analysis. The color gradient in (c) and (d) indicates vegetation density, from low (red) to high (green).

**Fig. S10**: Validation of environmental indices against ground truth ecological data for malacological survey sites. The figure uses violin plots to show the distribution of each index. Each ”violin” combines a box plot (inner white dot and black bar) with a kernel density plot (the outer shape), illustrating the median, interquartile range, and probability density of the data. The plots compare values for (a) NDVI, (b) NDWI, (c) MNDWI, and (d) EVI between marsh and non-marsh sites.

**Fig. S11**: Validation of environmental indices against ground truth ecological data for malacological survey sites. (a) NDVI by floating vegetation versus non-floating vegetation status. (b) NDWI by floating vegetation versus non-floating vegetation status. (c) MNDWI by floating vegetation versus non-floating vegetation status. (d) EVI by floating vegetation versus non-floating vegetation status.

**Fig. S12**: Distribution of Land Surface Temperature (LST) and Normalized Difference Vege-tation Index (NDVI) values across study villages. Each point represents a household location. The panels illustrate the much coarser spatial resolution of the MODIS LST data (1000m, left panel) compared to the Sentinel-2 NDVI data (10m, right panel).

**Fig. S13**: Comparison of the within-village coefficient of variation (CV) for Land Surface Tem-perature (LST) and NDVI.

**Fig. S14**: Comparison of model coefficient estimates for models using raw, unsmoothed NDVI (red) versus spatially smoothed NDVI (teal).

**Fig. S15**: Pairwise Spearman’s correlation matrix of all candidate predictor variables.

**Fig. S16**: Receiver operating characteristic (ROC) curve for the final malaria prediction model. Area under the curve (AUC) = 0.787.

**Fig. S17**: Joint interaction surface of age and environmental exposure. A contour showing the predicted probability of malaria infection (color scale) as a function of continuous age and NDVI

**Fig. S18**: Modeled joint effect of age and NDVI on malaria infection probability. 3D surface plot illustration of the predicted probability of malaria infection as a simultaneous function of age (years) and NDVI.

**Fig. S19**: Determinants of malaria infection with Tusting et al. home quality score. The forest plot shows adjusted odds ratios and 95% confidence intervals for predictors of malaria infection status from the sensitivity analysis with a binary home quality score by Tusting et al..

**Fig. S20**: Determinants of malaria infection of participants from 2023 and 2024 including bed net use. The forest plot shows adjusted odds ratios and 95% confidence intervals for predictors of malaria infection status from the sensitivity analysis with bed net use as a covariate.
